# Implementation and assessment of a structured curriculum for a 4-week pediatric rheumatology rotation for pediatric residents

**DOI:** 10.1186/s12909-024-05043-8

**Published:** 2024-01-23

**Authors:** Maynart Sukharomana, Sirirat Charuvanij

**Affiliations:** Division of Rheumatology, Department of Pediatrics, Faculty of Medicine Siriraj Hospital, Mahidol University, 2 Wanglang Road, 10700 Bangkoknoi, Bangkok, Thailand

**Keywords:** Confidence, Knowledge, Medical education, Pediatric residency, Pediatric rheumatology, Thai pGALS

## Abstract

**Background:**

General pediatricians often initially address children’s musculoskeletal (MSK) issues and play a crucial role in triaging and managing patients’ rheumatologic conditions. This study assessed the effectiveness of a structured curriculum in enhancing pediatric residents’ knowledge, MSK examination skills, and confidence during a 4-week pediatric rheumatology rotation.

**Methods:**

Pediatric residents in their either second or third year who participated in the 4-week rheumatology rotation once across three academic years (July 2020–June 2023) were enrolled. Residents’ knowledge, MSK examination skills, and confidence were assessed at pre- and post-rotation by using 25 multiple-choice questions, the Thai pediatric Gait Arms Legs Spine examination, and a questionnaire, respectively. The curriculum comprised instruction on MSK examinations, interactive lectures, case-based discussion, topic reviews, MSK radiology conference, clinical experience in rheumatology clinic and consultations, with self-guided learning with educational resources.

**Results:**

Fifty-eight pediatric residents (48 females, 10 males) with a mean age of 28.9 ± 0.8 years participated. Significant improvements were noted postrotation. Knowledge scores rose from 63.0 ± 12.2 to 79.7 ± 9.1 (mean difference 16.7 ± 10.3, *p* < 0.001). Similarly, MSK examination scores increased from 67.5 ± 14.4 to 93.6 ± 8.7 (mean difference 26.1 ± 14.6, *p* < 0.001). Residents also reported a marked increase in confidence across all evaluated areas, including history taking, MSK examination, arthrocentesis, and diagnosing and treating rheumatologic conditions (*p* < 0.001).

**Conclusions:**

The 4-week structured curriculum in the pediatric rheumatology rotation significantly enhanced pediatric residents’ knowledge, MSK examination skills, and confidence. These findings support the integration of pediatric rheumatology rotations into pediatric residency training programs.

**Supplementary Information:**

The online version contains supplementary material available at 10.1186/s12909-024-05043-8.

## Introduction

Pediatric rheumatology has emerged as a subspecialty in recent years, aiming to provide healthcare services for children with rheumatic diseases. However, a shortage of pediatric rheumatologists has led to inadequate pediatric rheumatology education and patient care [1, 2]. This has resulted in delays in diagnosis and treatment [3], potentially leading to disease-related damage and disability in patients [4]. In Southeast Asia and the Asia-Pacific regions, limited access to pediatric rheumatologists has necessitated the involvement of general pediatricians in the care of these patients [2]. However, studies have shown that pediatricians often lack confidence in pediatric rheumatology, particularly musculoskeletal (MSK) examination skills [5].

Given these challenges, enhancing pediatric rheumatology teaching during pediatric residency training is crucial. Training programs have implemented various methods to improve pediatric rheumatology education and confidence among learners. These include case-based discussions [6] and web-based teaching modules [7]. Additionally, online self-study resources such as the Pediatric Musculoskeletal Matters website are globally available to promote pediatric MSK education for physicians and allied health professionals [8].

In Thailand, the Royal College of Pediatricians of Thailand has incorporated pediatric rheumatology into the core syllabus of pediatric residency training. However, due to the country’s limited number of pediatric rheumatologists [9], formal teaching of pediatric rheumatology is not provided at all training institutes.

In the 2020 academic year, the Division of Rheumatology developed and introduced a new 4-week structured curriculum for pediatric residents. The Division of Rheumatology is one of the subspecialties in the Department of Pediatrics, Faculty of Medicine Siriraj Hospital, Mahidol University, Bangkok, Thailand. Our institute is the largest tertiary university-based hospital with 2,200 beds, which includes 303 beds for pediatric inpatient admissions, and provides the largest 3-year pediatric residency training program, with 26–28 pediatric residents in training for each academic year.

Our division developed the curriculum for the pediatric rheumatology rotation based upon Kern’s curriculum development model [10]. We used this model since it is widely used and accepted for curriculum development in medical education and provides a comprehensive systematic approach consisting of 6 steps including problem identification and general needs assessment, measurable goals, educational strategy, implementation, feedback and evaluation [10]. This curriculum aimed to enhance the pediatric residents’ knowledge and MSK examination skills in pediatric rheumatology. The objectives of the present investigation were to evaluate the impact of the structured curriculum on pediatric residents’ pediatric rheumatology knowledge, MSK examination skills, and confidence. We also explored the learners’ attitudes toward pediatric rheumatology and their expectations of the clinical rotation.

## Methods

### Setting and participants

This prospective single-center observational cohort study with pre-post study design of educational intervention was conducted at the Division of Rheumatology, Department of Pediatrics, Faculty of Medicine Siriraj Hospital, Mahidol University, Bangkok, Thailand.

The study was initiated in the 2020 academic year following the implementation of a new curriculum for the pediatric rheumatology rotation. The inclusion criteria consisted of pediatric residents in their second or third year of training who participated in the 4-week pediatric rheumatology rotation between July 2020 and June 2023, covering three consecutive academic years. Participants who could not complete the rotation activities or did not attend all the academic activities were excluded from the analysis.

### Curriculum structure

The development of the curriculum followed Kern’s six-step approach [10]. Problem identification and general needs assessments were based on our previous survey among 281 Thai residency-trained pediatricians [5]. The needs assessment of the specific target group was identified in a pilot group of current pediatric residents in training. The goals and objectives of the curriculum were to enhance knowledge of pediatric rheumatologic diseases, foster the development of pediatric MSK examination skills, and improve confidence in the clinical practice of pediatric rheumatology.

Regarding the pediatric rheumatology rotation, on the first day, pediatric residents underwent a pretest consisting of 25 multiple-choice questions covering various topics in pediatric rheumatology (with a total score of 100). Additionally, a questionnaire was administered to evaluate their experience and level of confidence in pediatric rheumatology clinical practice using a five-point Likert scale ranging from “very low” to “very high.” In the questionnaire, previous experience regarding the number of encountered cases with rheumatology chief complaints were self-reported by pediatric residents considering the average number of cases on a monthly basis.They also engaged in a self-study session using the pediatric Gait Arms Legs Spine (pGALS) tool [11]. 

During the first week of the rotation, a pretest of MSK examination skills was conducted at the pediatric rheumatology clinic, utilizing 18 items from the Thai pGALS [12]. Each item was scored 0 (not done), 1 (incomplete), or 2 (complete), with a total score converted to 100. MSK examination skills were taught by a pediatric rheumatologist who served as the teaching attending physician.

Educational activities throughout the rotation included two interactive academic lecture sessions (“Approach to Arthritis” in the second week and “Emergency in Pediatric Rheumatology” in the third week). There were also four weekly topic reviews on pediatric rheumatology, four sessions on case-based discussions, and an MSK radiology conference. These were complemented by four sessions in the rheumatology clinic and participation in inpatient and outpatient pediatric rheumatology consultations. Pediatric residents were provided with self-study materials through the Pediatric Musculoskeletal Matters website (www.pmmonline.org), the pGALS application [8, 13], and pediatric rheumatology core lectures accessible via the faculty’s online platform.

**Fig. 1:**
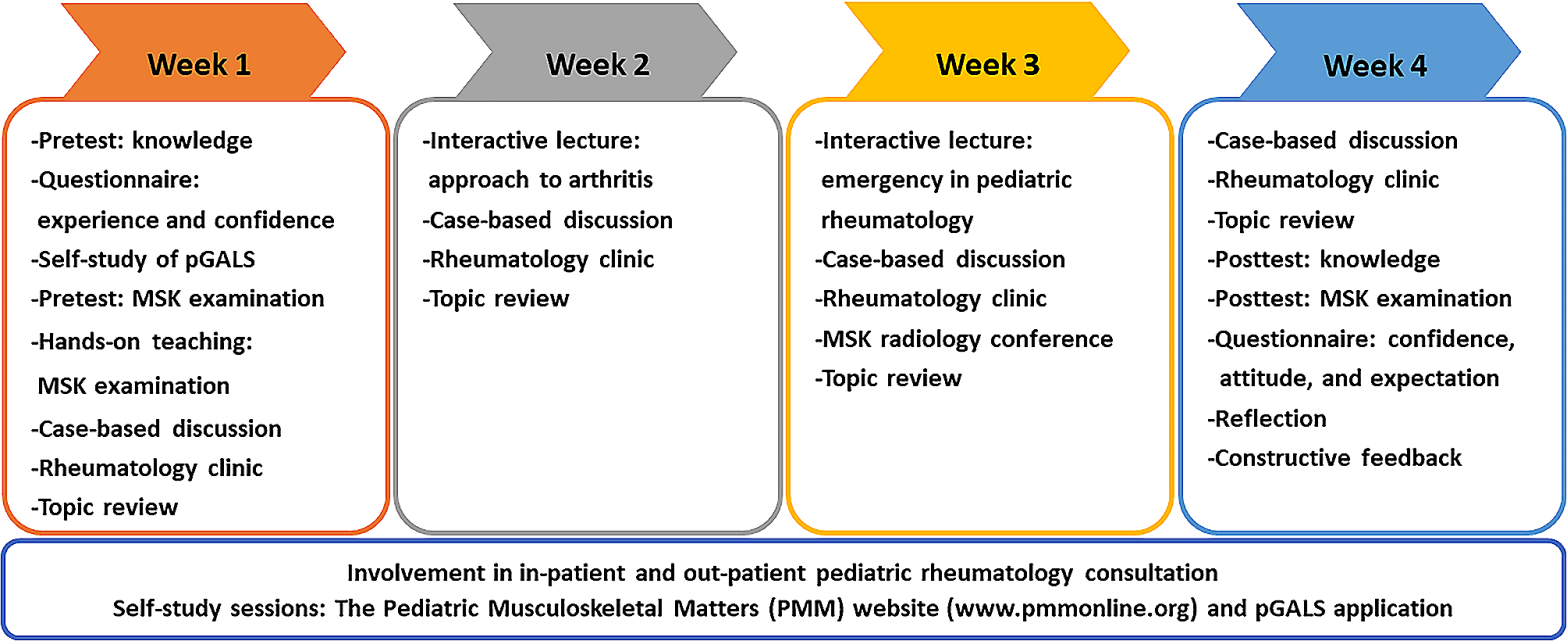
Schematic representation of the structured educational activities within the 4-week pediatric rheumatology rotation curriculum

At the end of the 4-week rotation, pediatric residents were reevaluated through posttests and an exit questionnaire to assess their confidence and attitude toward pediatric rheumatology. This questionnaire was adapted from a validated questionnaire previously used in a study involving residency-trained pediatricians [5]. In our exit questionnaire, a five-point Likert scale ranging from “very low” to “very high” was used to assess the level of confidence in pediatric rheumatology clinical practice, and a five-point Likert scale ranging from “strongly disagree” to “strongly agree” was used to assess the learners’ attitude and expectations toward pediatric rheumatology at the end of the rotation. Participants were also provided the opportunity to write free text for self-reflections such as what they have learned, what areas they want to learn more or improve, and suggestions to improve teaching in the clinical rotation. In addition to the questionnaires, the learners’ expectations regarding the pediatric rheumatology clinical rotation were explored through direct interviews. A feedback and reflection session was conducted at the end of each rotation. The information from participants will be used as the input for potential curriculum development following this study. The structured curriculum development and the educational activities implemented during the 4-week pediatric rheumatology rotation are depicted in Fig. 1. The curriculum mapping for the pediatric rheumatology rotation with details in each academic activity/intervention can be viewed in the Supplementary Table.

### Outcomes and measurements

The primary outcome variables assessed in this study included the pretest and posttest scores for pediatric rheumatology knowledge, MSK examination skills, and level of confidence in pediatric rheumatology practice. The intervention implemented was the structured pediatric rheumatology curriculum and the academic activities provided during the 4-week clinical rotation. This approach aimed to mitigate potential confounders that could affect residents’ competency, such as variations in the types and levels of clinical experiences that residents may encounter during their rotations.

This study was conducted in accordance with the principles outlined in the Declaration of Helsinki. Authorization for the study was obtained from the Siriraj Institutional Review Board of the Faculty of Medicine Siriraj Hospital, Mahidol University (approval number Si384/2020). All participants provided written informed consent before participating in the study. A research associate was involved in the process of obtaining written informed consent throughout the study period to avoid potential undue influence among the trainees.

### Sample size

As previous studies focusing on pediatric rheumatology education and/or MSK education did not directly assess the outcome by using pretest, posttest, or confidence scores, we could not calculate the sample size by using effect size differences. Therefore, the sample size for this study was determined by the convenience sampling method by considering the anticipated number of pediatric residents attending the rotation at our institute. At our institute, each academic year comprised 13 rotations, with the expectation that there would be at least one pediatric resident per rotation. Therefore, we estimated a minimum of 13 participants per academic year. To ensure an adequate sample size, we enrolled participants over three consecutive academic years, resulting in a total sample size of 39 participants or more, given the limited number of trainees available.

### Statistical analysis

All data analyses were conducted using PASW Statistics (version 18, SPSS Inc, Chicago, IL, USA). Descriptive statistics were utilized to summarize the participants’ demographic data and baseline clinical experiences, with results presented as counts and percentages. Continuous data comparisons are expressed as the means ± standard deviations (SDs) or as the medians and interquartile ranges, and comparisons were made using paired *t* tests or Mann–Whitney U tests, as appropriate. Categorical data comparisons were performed using the chi-square or Fisher’s exact tests. Ordinal data comparisons were conducted using the Wilcoxon signed-rank test. Independent sample *t* tests were employed to compare test scores between resident years 2 and 3, while a one-way analysis of variance was used to compare test scores between academic years. The threshold for statistical significance in this study was set at a *p* value of less than 0.05.

The reporting guidelines of the Strengthening the Reporting of Observational Studies in Epidemiology [14] were followed.

## Results

A total of 60 pediatric residents were initially enrolled in the study. However, 2 residents had to be excluded from the analysis due to medical illnesses that prevented them from participating in all activities during their 4-week rotation. Therefore, the final sample included 58 pediatric residents: 48 females (82.5%) and 10 males (17.5%). Their mean ± SD age was 28.9 ± 0.8 years. In terms of pediatric residency training, 38 residents (65.5%) were in their second year, while 20 residents (34.5%) were in their third year.

Regarding baseline clinical experiences, most residents (75.9%) reported having encountered 1 to 5 pediatric rheumatology cases per month. Only 3 residents (5.2%) reported encountering 6 to 10 cases, while 11 residents (19%) had never experienced pediatric rheumatology cases. The most common presenting manifestations observed by residents were joint pain (70.7%), followed by fever with unknown cause (65.5%), skin rash (53.4%), multiple organ involvement (44.8%), limb pain (25.9%), and limping (15.5%).

**Fig. 2:**
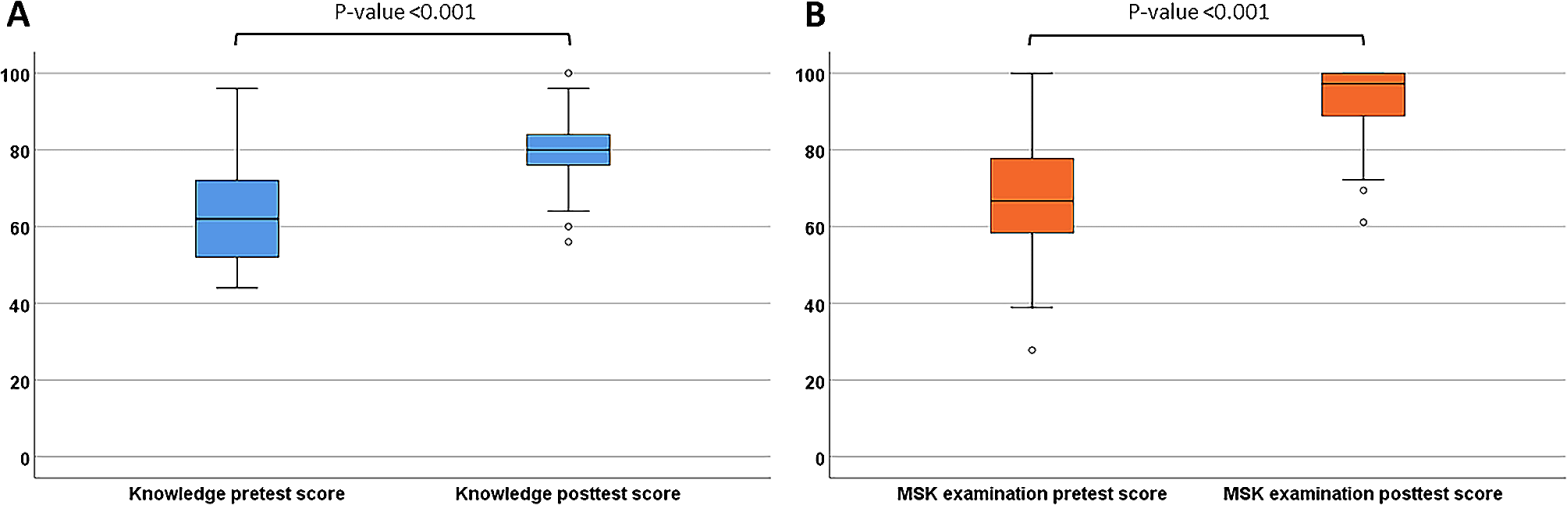
Analytical comparison of pediatric residents’ knowledge in pretests and posttests **(A)** and MSK examination skills in pretests and posttests **(B)** during the pediatric rheumatology rotation. There was a significant enhancement in the posttest performance (*p* < 0.001)

The mean ± SD posttest scores for knowledge and MSK examination skills showed a significant increase compared to the pretest scores (*p* < 0.001) as shown in Table 1; Fig. 2A and B. No significant differences were observed in the pretest and posttest scores when comparing subgroups based on resident years 2 and 3 or between academic years.

**Table 1:** Pretest and posttest assessments of pediatric rheumatology knowledge and musculoskeletal examination proficiency among participating pediatric residents (*N* = 58).

Test score	Pretest score Mean (± SD)	Posttest score Mean (± SD)	Mean difference	*p*	Paired samples correlation	*p*
**Pediatric rheumatology knowledge** (Score = 100)						
All participants (*N* = 58)	63.0 (± 12.2)	79.7 (± 9.1)	16.7 (± 10.3)	< 0.001	0.565	< 0.001
Resident year 2 (*n* = 38)	63.3 (± 13.0)	79.3 (± 9.6)	16.0 (± 10.6)	< 0.001	0.600	< 0.001
Resident year 3 (*n* = 20)	62.2 (± 10.7)	80.2 (± 8.4)	18.0 (± 9.9)	< 0.001	0.486	0.03
Academic year 2020 (*n* = 17)	64.0 (± 13.3)	81.6 (± 6.8)	17.6 (± 11.6)	< 0.001	0.488	0.047
Academic year 2021 (*n* = 23)	62.3 (± 12.7)	80.2 (± 9.8)	17.9 (± 10.2)	< 0.001	0.619	0.002
Academic year 2022 (*n* = 18)	62.9 (± 11.0)	77.1 (± 10.2)	14.2 (± 9.3)	< 0.001	0.616	0.007
**MSK examination skills** (Score = 100)						
All participants (*N* = 58)	67.5 (± 14.4)	93.6 (± 8.7)	26.1 (± 14.6)	< 0.001	0.276	0.036
Resident year 2 (*n* = 38)	68.5 (± 14.7)	93.8 (± 7.4)	25.3 (± 15.0)	< 0.001	0.215	0.196
Resident year 3 (*n* = 20)	65.8 (± 14.0)	93.4 (± 10.9)	27.6 (± 14.1)	< 0.001	0.375	0.103
Academic year 2020 (*n* = 17)	64.2 (± 16.5)	92.5 (± 8.9)	28.3 (± 17.4)	< 0.001	0.165	0.526
Academic year 2021 (*n* = 23)	69.7 (± 12.8)	95.7 (± 9.0)	26.0 (± 14.9)	< 0.001	0.092	0.676
Academic year 2022 (*n* = 18)	68.1 (± 14.4)	92.3 (± 8.1)	24.2 (± 11.5)	< 0.001	0.603	0.008

Abbreviations: IQR, interquartile range; MSK, musculoskeletal; SD, standard deviation

**Fig. 3:**
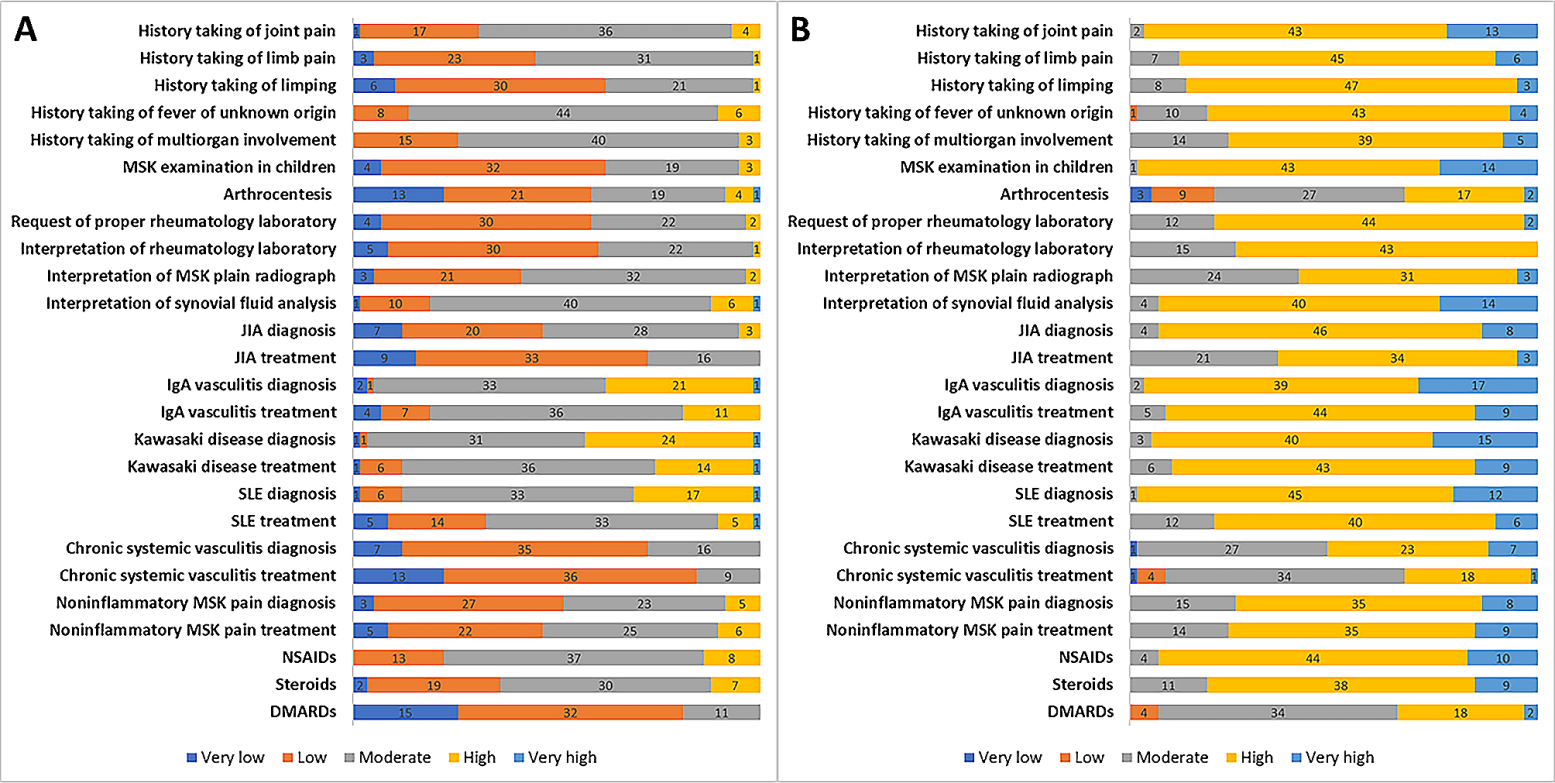
Confidence levels in various pediatric rheumatology topics reported by residents at the commencement **(A)** versus the conclusion **(B)** of the rotation. There was a significant increase in confidence levels at the end of the rotation (*p* < 0.001). Abbreviations: DMARDs, disease-modifying antirheumatic drugs; IgA vasculitis, immunoglobulin A vasculitis; JIA, juvenile idiopathic arthritis; MSK, musculoskeletal; NSAIDs, nonsteroidal anti-inflammatory drugs; SLE, systemic lupus erythematosus

The level of confidence in pediatric rheumatology clinical practice significantly increased across various aspects at the end of the rotation compared to the beginning of the rotation. These aspects include the history taking of rheumatologic and MSK diseases, MSK examination in children, arthrocentesis, rheumatology laboratory request and interpretation, interpretation of MSK plain radiographs, synovial fluid analysis, diagnosis and treatment of rheumatologic diseases, and common medication use in rheumatologic diseases such as nonsteroidal anti-inflammatory drugs, disease-modifying antirheumatic drugs, and corticosteroids. The *p* value for these improvements was < 0.001. Figure 3 A and 3B visually represent the level of confidence in each topic of pediatric rheumatology at the beginning and end of the pediatric rheumatology rotation.

**Fig. 4:**
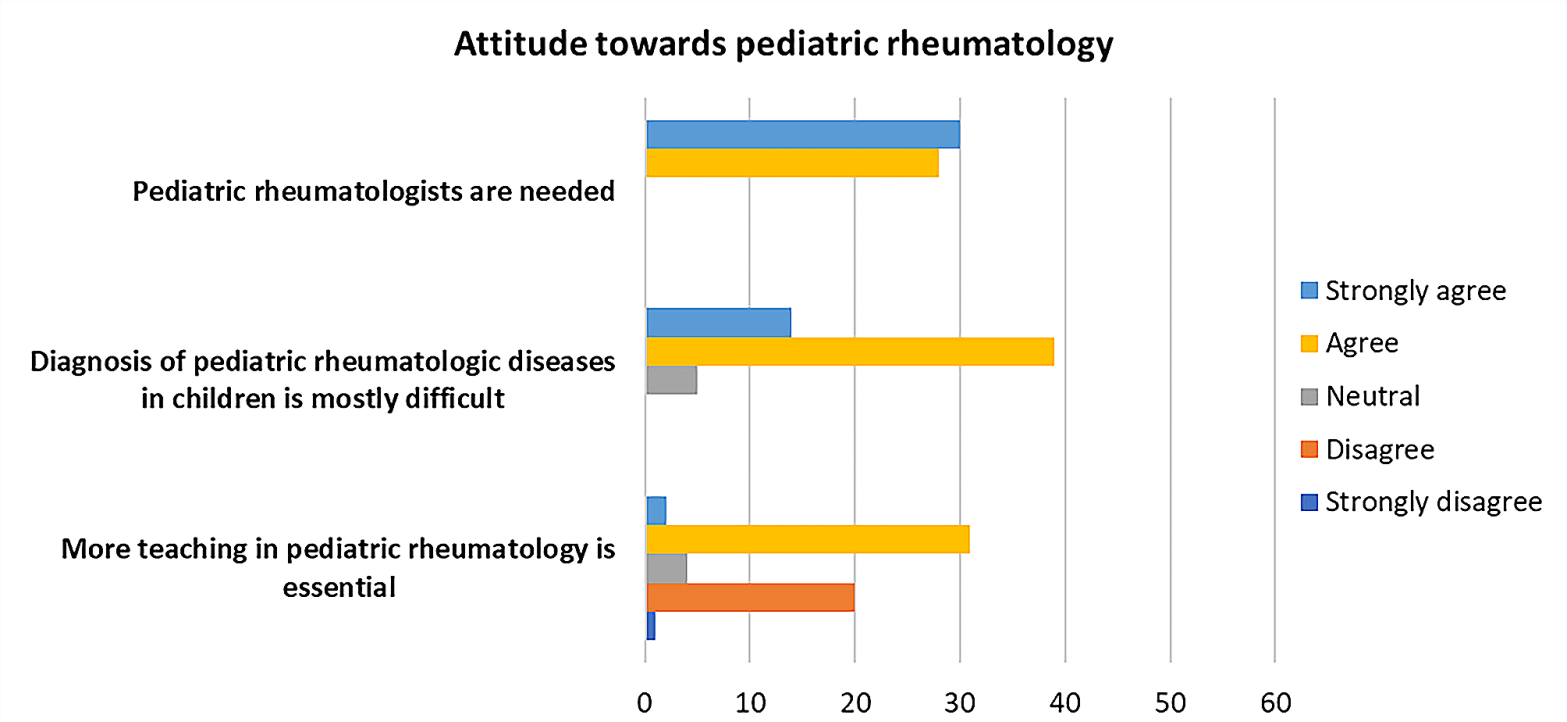
Survey insights into pediatric residents’ attitudes toward pediatric rheumatology (*N* = 58)

The exit survey aimed to gather the attitudes and expectations of pediatric residents regarding pediatric rheumatology and pediatric rheumatology education. More than half of pediatric residents (33, 56.9%) agreed or strongly agreed that more teaching in pediatric rheumatology during pediatric residency training is essential. As for participants who disagreed with this statement, it was found that they thought that what was being taught during the study period was optimal and adequate for the residency training program without the need to intensify more teaching than what was already provided. The vast majority of pediatric residents (53, 91.4%) expressed that diagnosing pediatric rheumatologic diseases is mostly difficult. Furthermore, all pediatric residents (58, 100%) agreed that pediatric rheumatologists are needed. Figure 4 illustrates the attitudes of pediatric residents toward pediatric rheumatology at the end of the rotation.

Regarding expectations for pediatric rheumatology education, most residents preferred lectures (40, 69.0%), case-based discussions (35, 60.3%), and workshops (33, 56.9%) as the preferred teaching methods. The most commonly requested teaching topics were knowledge and management in pediatric rheumatologic diseases (55, 94.8%) and arthrocentesis (54, 93.1%). Additionally, the residents suggested the teaching topics of multisystem inflammatory syndrome in children, uveitis, and disease-modifying antirheumatic drugs.

## Discussion

The structured curriculum implemented during the 4-week pediatric rheumatology rotation significantly impacted pediatric residents’ competency. The context of the pediatric residency training program provides predominantly both outpatient and inpatient clinical exposure, which also applies to context of the pediatric rheumatology clinical rotation. The curriculum led to improvements in both their knowledge of pediatric rheumatologic diseases and their MSK examination skills. Additionally, the rotation resulted in a notable increase in confidence among residents in various aspects of pediatric rheumatology clinical practice. The exit survey findings further emphasized the importance of pediatric rheumatology teaching, with a majority of residents agreeing on its essentiality. Among the requested teaching topics, management in pediatric rheumatic diseases and arthrocentesis were the two most commonly mentioned areas of interest.

Our study demonstrated that a structured curriculum incorporating a combination of teaching methods yielded positive outcomes for pediatric residents. The curriculum included interactive academic lectures, hands-on demonstrations, case-based discussions, MSK radiology conferences, topic reviews, and self-directed learning. These methods facilitated the development of cognitive and psychomotor skills, as well as the cultivation of positive attitudes toward pediatric rheumatology [5–8, 13]. Previous research has shown that case-based studies are a favored option for pediatricians [5]. This teaching method has proven beneficial during residency training [6] as the studied cases are drawn from real-life practice. Our pediatric residents were also assigned cases to review, and they discussed the tentative management plans with their supervising attending physician a day before attending the rheumatology clinic.

The 4-week pediatric rheumatology rotation had a daily schedule starting from 9 A.M. to 4 P.M. on weekdays. All activities and assessment were accommodated within these working hours. Time slots specifically for self-study were allocated. Pediatric residents may also access self-study online platforms at available time slots during the working hours or afterwards. Pediatric residents also participated in morning inpatient rounds of pediatric rheumatology patients along with the fellows on weekdays and on alternate weekends. Although this curriculum may have some similarities with other subspecialty rotations such as outpatient and inpatient consultation, attendance of outpatient clinic, topic reviews, the uniqueness of the pediatric rheumatology curriculum is its well-structured combination with various forms of teaching methods with intervention and assessment of outcomes for knowledge, skills, and confidence in clinical practice, as well as providing work-life balance for the trainees.

It is widely acknowledged that clinical exposure plays a crucial role in developing competency [15]. Workplace-based learning is fundamental in postgraduate teaching; however, the type of cases and conditions that learners encounter during their clinical practice can vary unpredictably, leading to differences in their learning experiences. To address the rarity of certain emergency conditions in rheumatology, we incorporated interactive academic lectures to cover essential conditions such as macrophage activation syndrome, catastrophic antiphospholipid syndrome, pulmonary-renal syndrome, and scleroderma renal crisis. This approach aimed to minimize variations in learning content resulting from chance clinical exposure. On the other hand, pediatric rheumatologic diseases such as juvenile idiopathic arthritis and systemic lupus erythematosus are prevalent in rheumatology clinics and inpatient consultation services [16]. In our study, we ensured consistent educational activities throughout each rotation in the academic year. Thus, the 4-week rotation provided sufficient clinical exposure to these conditions.

The pGALS screening tool, developed by Foster et al., is valuable for screening MSK abnormalities in children [11]. It has proven to be practical and applicable in various settings, including outpatient departments [12], pediatric acute care [17], and sports medicine [18]. One of the advantages of the pGALS tool is that it can be performed by individuals who are not MSK medicine or rheumatology experts [17]. The original English version of the pGALS tool has been translated into multiple languages and has gained widespread use worldwide [12, 19–23]. In our previous study, we linguistically validated the Thai version of the pGALS and confirmed its validity [12]. Our findings demonstrated that the Thai pGALS is not only beneficial for pediatric residents in detecting MSK abnormalities in children but also practical and well received by patients and their parents [12]. Based on these results, we incorporated formal teaching of the Thai pGALS into the curriculum development for our study. Our study also stressed the impact of formal hands-on teaching and practice of MSK examination using the pGALS tool. The results indicated that pediatric residents showed a significant improvement in MSK examination skills at the end of the rotation compared to the beginning. This finding suggests that providing formal instruction and practical training in MSK examination enhances skills more effectively than having residents rely solely on self-study with provided resources.

There has been limited research on medical education in pediatric rheumatology within the context of pediatric residency training. Gillispie et al. conducted a study that demonstrated the effectiveness of case-based discussions in improving the confidence and knowledge of pediatric residents in pediatric rheumatology [6]. Similarly, Batthish et al. found that web-based teaching modules, particularly those using case-based approaches and multimedia modalities, were valuable in teaching MSK examination methods to pediatric residents [7]. However, our study revealed a preference among pediatric residents for academic lectures rather than case-based discussions. This preference may reflect differences in individual learning styles and educational cultures between Asian and Caucasian trainees. Nevertheless, it is essential to note that according to the National Training Laboratories learning pyramid model, the student retention rate for information delivered through lectures is only 5% [24]. Furthermore, a survey study involving pediatricians demonstrated that attending academic pediatric rheumatology lectures did not significantly increase their confidence in clinical practice skills [5].

Various innovative teaching methods have become available, such as flipped classrooms, interprofessional education, team-based learning, gamification, and augmented reality [25–28]. Whether these novel approaches can be effectively applied to pediatric rheumatology remains to be seen, and further research is needed in this area. Additionally, virtual learning methods became especially beneficial during the Coronavirus disease 2019 pandemic [28]. Our program was modified in response to the pandemic so that some educational activities were delivered through the Zoom platform.

The Pediatric Rheumatology European Society has developed educational portfolios to enhance the knowledge of medical professionals in providing care for children with rheumatic diseases [8]. Additionally, the Pediatric Musculoskeletal Matters website (www.pmmonline.org*)* is a valuable online resource accessible worldwide to physicians and allied health professionals [8, 13]. This resource has the potential to benefit pediatricians in their practice. Therefore, we strongly encourage pediatric residents to not only utilize these valuable educational resources during their self-directed learning sessions but also to continue engaging in lifelong learning by using them.

The development of clinical competencies, as proposed by Miller in 1990 [29], follows a hierarchical pyramid model consisting of four processes. They are Level 1, “knows (knowledge)”; Level 2, “knows how (competence)”; Level 3, “shows how (performance)”; and Level 4, “does (action).” Each level can be assessed using different methods [30]. In our study, we employed multiple-choice questions to assess knowledge and the Thai pGALS tool for the performance of MSK examination through direct observation in the rheumatology clinic. Note that the posttests were conducted at the end of the rotation, reflecting short-term retention. When assessing competencies in medical education, long-term retention should also be evaluated [31]. It can be assessed through formative and summative examinations, such as multiple-choice questions, constructed response questions, and objective structured clinical examinations for the residency board examination. Additionally, workplace-based assessments using entrustable professional activities at different milestones during each academic level offer several benefits in medical education, such as feedback-seeking stimulation [32]. While our institute has implemented all of these assessment methods during training, they are beyond the scope of this study and will not be discussed in this context.

The level of confidence in pediatric rheumatology clinical practice significantly increased from the beginning to the end of the rotation. In a study conducted by Chowichien et al., pediatricians reported low confidence in MSK examination, arthrocentesis, and interpretation of rheumatology investigations [5]. Interestingly, pediatricians who had received training from pediatric rheumatology specialists during their residency showed a relatively higher confidence level in MSK examination and arthrocentesis than those who did not receive such training [5]. Previous studies have also highlighted low confidence in MSK examination among trainee pediatricians [33] and physicians from other specialties [34]. In our study, most residents initially reported a low level of confidence in the MSK examination methods at the beginning of the rotation but demonstrated a high to very high level of confidence by the end of the rotation. This marked improvement indicates that formal teaching of MSK examination and utilizing the pGALS tool are effective in improving residents’ confidence in MSK examination as well as their performance. Similarly, a study by Boulter et al. demonstrated the benefit of pGALS in enhancing confidence in MSK examination among junior doctors [35].

When assessing confidence in arthrocentesis, most residents in our study initially reported a low level of confidence at the beginning of the rotation, which improved to a moderate level by the end of the rotation. Although the statistical analysis showed a significant increase, the modest increase in confidence highlights the need to further enhance the formal teaching of arthrocentesis. Using a knee model for practicing arthrocentesis has been shown to be beneficial in improving confidence and performance among sixth-year medical students [36]. Therefore, it is recommended to introduce innovative methods such as the use of models or simulations to facilitate arthrocentesis skills during residency training.

The level of confidence in diagnosing and treating pediatric rheumatic diseases among pediatricians can vary depending on the specific diseases [5]. In our study, at the beginning of the rotation, pediatric residents reported a moderate level of confidence in diagnosing juvenile idiopathic arthritis but a low level of confidence in its treatment. This discrepancy could be attributed to their previous experiences in encountering children with joint pain, which is a common presenting symptom, and their familiarity with this condition. However, after attending the rotation, most residents reported high confidence in both the diagnosis and the treatment of juvenile idiopathic arthritis. For chronic systemic vasculitis, most residents reported an increase in confidence from low at the beginning to moderate at the end of the rotation. Limited exposure to these less common conditions during clinical training likely contributed to their initial lack of confidence. However, given the complexity of pediatric rheumatic diseases, it is recommended that early referral to specialists for further evaluation and proper management be emphasized. In real-life practice, general pediatricians are often the first to encounter these patients [2]. Therefore, during residency training, emphasis should be placed on developing competency in disease knowledge, MSK examination skills, emergency management, initial treatment, and early referral to specialists.

Several limitations in our study should be acknowledged. First, it is important to recognize the potential confounding factors that may have influenced the participants’ competency levels. Variations in clinical exposures by chance could have differed between participants, leading to potential bias. However, we attempted to mitigate this by implementing the same structured learning content and educational activities in each rotation. Additionally, the identified areas of educational needs likely reflect the residents’ clinical exposure, and thus may be different in other pediatric residency training programs with various contexts. As this is a single-center study, further studies to explore the educational needs in other pediatric residency training institution should be explored. Second, assessing competency through posttests within the 4-week rotation may only partially capture the long-term retention of knowledge and skills. While the assessment methods used to evaluate competencies and skills have been assessed as part of the pediatric residency training program, they were not explicitly evaluated within the context of this study. In this study, we did not repeat the tests and questionnaire to assess the long-term knowledge retention and confidence, nor were there formal surveys of routine pGALS examination after attending the rotation. However, these assessment methods would be of great benefit to evaluate long-term retention, and should be considered to perform at 6 or 12 months in further studies. Third, there could be possibilities to overestimate the effects of interventions and underreport of unintended consequences of the curriculum as participants who cannot complete the rotation or did not attend all activities were excluded from the analysis. To accommodate these scenarios which may occur in real life settings, we provided the opportunity for the trainees to catch up with the missed educational activities later, not limited to within the 4-week rotation, for their benefit in pediatric rheumatology education. Last, the number of participants in our study was limited even though we recruited pediatric residents over three academic years. However, it is worth noting that our center has the largest pediatric residency training program in Thailand and is the only center with year-round arrangements for residents rotating in pediatric rheumatology. Despite these limitations, the findings of our study may have potential applicability to other institutes aiming to develop a structured curriculum with educational activities within their general pediatric residency training programs.

## Conclusions

The 4-week structured curriculum in the pediatric rheumatology rotation significantly increased pediatric residents’ knowledge, MSK examination skills, and confidence. Based on these findings, we strongly recommend including a pediatric rheumatology rotation in residency training programs. The structured curriculum and educational activities are a potential model for teaching pediatric rheumatology during residency training.

### Electronic supplementary material

Below is the link to the electronic supplementary material.

Supplementary Material 1: The curriculum mapping for the pediatric rheumatology rotation with details in each academic activity/intervention.

## Data Availability

The datasets used and/or analysed during the current study are available upon reasonable request by contacting the corresponding author.

## Abbreviations

DMARDs: Disease-modifying antirheumatic drugs
IgA vasculitis: Immunoglobulin A vasculitis
IQR: Interquartile range
JIA: Juvenile idiopathic arthritis
MSK: Musculoskeletal
NSAIDs: Nonsteroidal anti-inflammatory drugs
pGALS: Pediatric Gait Arms Legs Spine
SD: Standard deviations
SLE: Systemic lupus erythematosus
