# Correction: Implementation and assessment of a structured curriculum for a 4-week pediatric rheumatology rotation for pediatric residents

**DOI:** 10.1186/s12909-024-05190-y

**Published:** 2024-02-28

**Authors:** Maynart Sukharomana, Sirirat Charuvanij

**Affiliations:** Division of Rheumatology, Department of Pediatrics, Faculty of Medicine Siriraj Hospital, Mahidol University, 2 Wanglang Road, 10700 Bangkoknoi, Bangkok, Thailand

**Correction: BMC Medical Education (2024) 24:83**

10.1186/s12909-024-05043-8

Following publication of the original article [1], the authors would like to correct the Results section.

The sentence currently reads:

The vast majority of pediatric residents (53, 91.4%) agreed or strongly agreed that more teaching in pediatric rheumatology during pediatric residency training is essential.

Approximately half of the pediatric residents (31, 53.4%) expressed that diagnosing pediatric rheumatologic diseases is mostly difficult.

The sentence should read:

More than half of pediatric residents (33, 56.9%) agreed or strongly agreed that more teaching in pediatric rheumatology during pediatric residency training is essential.

The vast majority of pediatric residents (53, 91.4%) expressed that diagnosing pediatric rheumatologic diseases is mostly difficult.

The original article has been corrected.
