# Supplementary material for: Implementation and assessment of a structured curriculum for a 4-week pediatric rheumatology rotation for pediatric residents
Source: BMC Med Educ. 2024 Jan 23;24:83. doi: 10.1186/s12909-024-05043-8 (PMC10804586; doi:10.1186/s12909-024-05043-8)
Supplement: Supplementary file 1 — Supplementary Material 1: The curriculum mapping for the pediatric rheumatology rotation with details in each academic activity/intervention. [file 12909_2024_5043_MOESM1_ESM.docx]

**Supplementary Table.**  The curriculum mapping for the pediatric rheumatology rotation with details in each academic activity/intervention

| **Activities/**  **Interventions** | **Inputs** | | | **Process** | | **Assessment tool** | **Output/**  **outcome** |
| --- | --- | --- | --- | --- | --- | --- | --- |
|  | **Contents** | **Manpower** | **Resource** | **Teaching/**  **learning mode** | **Hours(H),**  **Week(W)** |  |  |
| Knowledge | | | | | | | |
| Pretest: knowledge in pediatric rheumatology | Essential knowledge in pediatric rheumatology | Attending physician (MS, SC) designed the MCQ, supervised test | Textbook of Pediatric Rheumatology*,  pmmonline.org, online department lectures platform (asynchronous) | Paper-based 25 items MCQ | 0.5 H/4 W  (W1) | Paper-based 25 items MCQ pretest | Knowledge in pediatric rheumatology |
| Case-based discussion (Flipped classroom) | Patient chart reviews with discussions | Attending physician  (MS, SC)  facilitated discussions | Textbook of Pediatric Rheumatology*, pmmonline.org, online department lectures platform (asynchronous) | Case-based discussion (Flipped classroom) | 2 H/W  (W1-4) | Entrustable professional activities assessment (beyond the context of this research) and direct observation | Knowledge in pediatric rheumatology diseases |
| Topic review | Topics in pediatric rheumatology selected for review | Attending physician (MS, SC) facilitated discussions | Journal articles,  Textbook of Pediatric Rheumatology* | Learner-centered, hybrid platform (onsite, online Zoom®) | 1 H/W  (W1-4) | Paper-based 25 items MCQ posttest  end of rotation and direct observation | Knowledge in pediatric rheumatology |
| Interactive lecture: approach to arthritis | Principles of history taking, physical examination, diagnosis, common diseases | Attending physician (MS) taught the lecture | Textbook of Pediatric Rheumatology*,  pmmonline.org, pGALS  application,  journal articles | Interactive lecture | 1 H/4 W  (W2) | Paper-based 25 items MCQ posttest  end of rotation | Knowledge in approach to arthritis in children |
| Interactive lecture:  emergency in pediatric  rheumatology | Emergency conditions in pediatric rheumatology | Attending physician (SC) taught the lecture | Textbook of Pediatric Rheumatology*,  pmmonline.org,  journal articles | Interactive lecture | 1 H/4 W  (W3) | Paper-based 25 items MCQ posttest  end of rotation | Knowledge in emergency problems in pediatric rheumatology |
| MSK radiology conference | 3 case discussions with MSK imaging | Attending physician (MS, SC) facilitated discussions with MSK radiology specialist | MSK radiology consultation | Interactive case discussion | 1 H/4 W  (W3) | Paper-based questionnaire  5 Likert scale at the end of rotation for confidence in plain radiograph interpretation | Knowledge in MSK radiology applicable to rheumatology and principles of plain radiograph interpretation |
| Self-study sessions | Common problems in pediatric rheumatology | Self-study by trainees | pmmonline.org, online department lecture platform (asynchronous) | Online platforms (asynchronous) self-paced learning | 3 H/W  (W1-4) | - | Knowledge in pediatric rheumatology |
| Posttest: knowledge in pediatric rheumatology | Essential knowledge in pediatric rheumatology | Attending physician (MS, SC) designed the MCQ, supervised test | Textbook of Pediatric Rheumatology*,  pmmonline. org website,  online department lecture platform (asynchronous) | Paper-based 25 items MCQ | 0.5 H/4 W  (W4) | Paper-based 25 items MCQ posttest  end of rotation | Knowledge in pediatric rheumatology |
| Skills | | | | | | | |
| Self-study of pGALS | Thai pGALS MSK examination steps | Self-study by trainees in allocated time slot | pmmonline.org, pGALS application, Thai pGALS,  pGALS VDO | Online platforms (asynchronous) self-paced learning | 2 H/4 W  (W1) | Pretest for MSK exam using Thai pGALS checklist | Skills in MSK examination |
| Pretest: MSK examination | Thai pGALS MSK examination steps | Attending physician (MS, SC) designed the checklist, supervised test | Thai pGALS | Direct observation | 0.5 H/4 W  (W1) | Thai pGALS checklist 18 items (assess as complete, incomplete, not done for each item) | Skills in MSK examination |
| Hands-on teaching: MSK examination | Thai pGALS MSK examination steps | Attending physician (MS, SC) taught MSK examination | Thai pGALS, pGALS application, pmmonline.org,  pGALS VDO | Hands-on teaching at clinic | 0.5 H/4 W  (W1) | Thai pGALS checklist and step by step MSK exam maneuvers | Skills in MSK examination |
| Rheumatology clinic | Pediatric rheumatology diseases (e.g. JIA, CTD, vasculitis, non-inflammatory MSK pain, postinfectious arthritis, MIS-C) | Attending physician (MS, SC) provided consultation with management in each patient | Textbook of Pediatric Rheumatology*,  pmmonline.org, online department lecture platform (asynchronous) | Consultation and interactive case discussion with attending physician | 3 H/W  (W1-4) | Entrustable professional activities assessment (beyond the context of this research) | Knowledge, clinical exposure to pediatric rheumatology patients and skills in MSK examination |
| Outpatient-Inpatient consultation and rounds | Pediatric rheumatology diseases | Attending physician (MS, SC) provided consultation with management in each patient | Textbook of Pediatric Rheumatology*,  pmmonline.org, online department lecture platform  (asynchronous) | Consultation and interactive case discussion with attending physician | 2 H/Day | Entrustable professional activities assessment (beyond the context of this research) | Knowledge, clinical exposure to pediatric rheumatology patients and skills in MSK examination |
| Posttest: MSK examination | Thai pGALS MSK examination steps | Attending physician (MS, SC) designed the checklist, supervised test | Thai pGALS | Direct observation at clinic | 0.5 H/4 W  (W4) | Thai pGALS checklist 18 items (assess as complete, incomplete, not done for each item) | Skills in MSK examination |
| Confidence | | | | | | | |
| Questionnaire at pre-rotation: experience and confidence | Assessment of previous experience in pediatric rheumatology and confidence in pediatric rheumatology clinical practice before the rotation | Attending physician (MS, SC) designed the question-naire | Adaptations from a questionnaire performed in Thai residency-trained pediatricians | Paper-based questionnaire  5 Likert scale | 0.5 H/4 W  (W1) | Paper-based questionnaire  5 Likert scale | Confidence in pediatric rheumatology clinical practice |
| Questionnaire at post-rotation: confidence, attitude, and expectation | Assessment of confidence in pediatric rheumatology clinical practice after attending 4-week rotation, attitude and expectation in pediatric rheumatology | Attending physician (MS, SC) designed the questionnaire | Adaptations from a questionnaire performed in Thai residency-trained pediatricians with addition of free-text reflection | Paper-based questionnaire  5 Likert scale | 0.5 H/4 W  (W4) | Paper-based questionnaire  5 Likert scale | Confidence in pediatric rheumatology clinical practice, attitude and expectation in pediatric rheumatology |
| Reflection | Self-reflection of what the residents have learned, what to learn more or improve, and suggestions to improve teaching | Attending physician (MS, SC) designed the free-text questionnaire | Kern’s 6 steps to curriculum development | Paper-based questionnaire for free-text writing, individual interview with  reflection | 0.5 H/4 W  (W4) | Assessment of reflection | Confidence in pediatric rheumatology clinical practice and reflection of clinical experiences in the rotation |
| Constructive feedback | Constructive feedback of the overall performance for each resident at end of rotation | Attending physician (MS, SC) provided constructive feedback | Kern’s 6 steps to curriculum development | Feedback session | 0.5 H/4 W  (W4) | Assessment of feedback | Confidence in pediatric rheumatology clinical practice and feedback of clinical experiences in the rotation |

*References for the Textbook of Pediatric Rheumatology include the following:

-Petty RE, Laxer RM, Lindsley CB, Wedderburn LR, Mellins ED, Fuhlbrigge RC, editors. Textbook of Pediatric Rheumatology. 8^th^ Edition. Philadelphia: Elsevier; 2021

-Petty RE, Laxer RM, Lindsley CB, Wedderburn LR, editors. Textbook of Pediatric Rheumatology: 7^th^ Edition. Philadelphia: Elsevier; 2016.
